# The association between *Helicobacter pylori* infection and the risk for gout in hyperuricemia patients in China – A cross-sectional study

**DOI:** 10.1186/s13099-024-00615-x

**Published:** 2024-04-27

**Authors:** Lin Chen, Yue Zhu, Yilin Huang, Keqing Shen, Liying Chen

**Affiliations:** Department of General Practice, Sir Run Run Shaw Hospital, School of Medicine, Zhejiang University, Hangzhou Zhejiang, China

**Keywords:** Gout, Hyperuricemia, *H. Pylori* infection, Cross-sectional study

## Abstract

**Purpose:**

*Helicobacter pylori* (*H. pylori*) infection has been reported to be associated with multiple metabolic diseases. However, the connection between *H. pylori* infection and gout has not been explored previously. Our study aimed to investigate the association of gout and *H. pylori* infection in hyperuricemia population in China.

**Patients and methods:**

This cross-sectional study was performed among the subjects who underwent health checkup in our health promotion center from January 1, 2020 to December 31, 2021. A total of 53,629 subjects with a mean age of 44.2 years were included in this study. *H. pylori* infection was defined as a positive [13]C-urea breath test. The effect of *H. pylori* infection on gout was assessed by multiple logistic regression analysis.

**Results:**

720 subjects with gout and 15,077 subjects with asymptomatic hyperuricemia (> 420 µmol/L in male and > 360 µmol/L in female) were enrolled. The prevalence rates of *H. pylori* infection, hyperuricemia and gout were 26.3%, 29.5%, 1.3%, respectively. The prevalence rate of *H. pylori* infection was significantly higher in subjects with gout than in those with asymptomatic hyperuricemia (35.0% vs. 27.2%; *P*<0.001). Multiple logistic regression analysis showed that *H. pylori* infection was associated with an increased risk of gout independent of serum uric acid level in hyperuricemia population (odds ratio [OR]: 1.320, 95% confidence interval [CI]: 1.124–1.550, *P* = 0.001).

**Conclusion:**

*H. pylori* infection is positively associated with higher risk of gout in hyperuricemia population. The causal relationship and potential mechanism between *H. pylori* infection and gout warrants further investigation.

## Introduction

*Helicobacter pylori* (*H. pylori*) is a gram-negative spiral bacterium that colonizes in the gastric mucosa persistently, infecting approximately 4.4 billion people worldwide and varying considerably according to geographic region [1]. *H. pylori* infection is associated to several conditions including gastric cancer, mucosa-associated lymphoid tissue (MALT) lymphoma, peptic ulcer disease, gastric atrophy, gastric intestinal metaplasia [2, 3]. Therefore, *H. pylori* was classified as a group 1 carcinogen leading to gastric adenocarcinoma by the World Health Organization (WHO) [2]. In recent decades, evidence also linked *H. pylori* infection with many extra-gastroduodenal conditions including hematological, metabolic, cardiovascular, neurodegenerative and allergic disorders [4, 5].

Gout is a chronic disease with periods of inflammation, known as flares, resulting from the deposition of monosodium urate crystals in joints, tendons and other structures [6, 7]. Population-based studies have reported the prevalence rate of gout ranges from 0·68% to 3·90% among different areas worldwide, due to the variation of ethnic groups and regions [8–12]. A meta-​analysis including 30 studies published from 2000 to 2016 found a pooled prevalence of gout of 1.1% in mainland China [13]. A more recent epidemiological survey estimated that the prevalence rate of gout among general Chinese adult population has reached 3.2%, corresponding to 25.56 million adults with gout during 2015–2017^14^. The increasing trend of the gout prevalence is possibly due to the aging population as well as the growing rates of metabolic diseases in recent years, which has imposed heavy burden on the global economy by causing substantial medical costs and productivity losses. Hospital admissions for gout have increased by 50–100% in the UK, USA, Canada and Sweden over recent decades [15–19]. Though high serum urate concentration is assumed to be the key risk factor for gout, a large proportion of hyperuricemia patients are exempt from developing gout through a lengthy follow-up period [13]. Up to 76% of asymptomatic hyperuricemia individuals fail to find monosodium urate (MSU) crystal deposition by dual energy CT scan of the feet [20], which triggers our interests on exploring the potential risk factors leading to the gout attack among the hyperuricemia individuals.

Several studies have reported a significant association between *H. pylori* infection and coronary heart disease, especially for the CagA-positive strains [4, 21, 22]. *H. pylori* + CagA + infection might be involved in coronary atherosclerosis via the change of serum lipids profile, enhancement of low-density lipoprotein cholesterol (LDL-C) oxidation, and activation of inflammatory responses [23]. Further study found that the high-density lipoprotein cholesterol (HDL-C) levels increased after *H. pylori* eradication, prompting an underlying benefit on cardiovascular diseases from *H. pylori* eradication [24]. Potential connections between *H. pylori* infection and metabolic diseases such as obesity, metabolic syndrome, insulin resistance, diabetes mellitus and nonalcoholic fatty liver disease (NAFLD) have also been indicated [5, 25]. Though the link between *H. pylori* infection and multiple metabolic diseases has been extensively explored, there is in lack of study investigating the possible association between *H. pylori* infection and gout.

Therefore, we conducted a cross-sectional study to investigate the relationship between gout and *H. pylori* infection in hyperuricemia population, which may provide evidence for screening and identifying the high-risk population of gout.

## Methods

### Study design and subjects

This cross-sectional study was conducted at the Health Promotion Center in Sir Run Run Shaw Hospital affiliated with Zhejiang University. A total of 56,471 participants who underwent a standard 13 C-urea breath test as part of their health checkup in the period from January 1, 2020 to December 31, 2021 were enrolled in this study.

Subjects meeting the following criteria were excluded:

1. Those with a self-reported history of gastric surgery (*n* = 99);
2. Those with a self-reported history of malignant tumor (*n* = 679);
3. Those with self-reported history of severe hereditary or auto-immune diseases (*n* = 754);
4. Those with missing data (*n* = 1262).

A total of 53,629 eligible subjects were finally enrolled (31,692 male and 21,937 female). This study was approved by the Ethics Committee of Sir Run Run Shaw Hospital, affiliated with Medical College of Zhejiang University (NO. 2023-0047).

### Data collection

Information including past medical history (including gout), alcohol consumption, cigarette smoking, family history, and physical examination were collected by well-trained general practitioners. Alcohol consumption and smoking status were classified as current (> 6 months on a daily basis) or non-current status.

Weight, height, blood pressure (BP), and waist circumference (WC) were measured by well-trained nurses. Standing height and body weight were measured without shoes or coats. Body mass index (BMI, kg/m^2^) was calculated as weight in kilograms divided by height in meters squared. BP was measured by an automated sphygmomanometer with the subject maintaining sitting position. WC was measured with the measuring tape positioned midway between the lowest rib and the iliac crest.

Fasting blood samples were obtained from an antecubital vein to be used for the analysis of biochemical values. Total cholesterol (TC), triglycerides (TG), HDL-C, LDL-C, total protein (TP), alanine aminotransferase (ALT), aspartate aminotransferase (AST), c-glutamyltransferase (GGT), alkaline phosphatase (ALP), total bilirubin (TBil), fasting glucose (FBG), HbA1c, urea nitrogen (BUN), creatinine (CR), and uric acid (UA) were measured after an 8-hour overnight fast.

### Diagnostic criteria for *H. pylori* infection

After overnight fasting for 8 h, all participants underwent a [13]C-urea breath test (UBT) in our Health Promotion Center. After collecting an initial baseline breath sample, participants took 75 mg of [13]C-urea that was dissolved in 200 mL 0.1 N of citric acid made up in water. The second breath sample was collected 30 minutes later. A difference between the baseline sample and the second sample that exceeded 3.5 parts per 1000 of [13]CO2 was defined as positive for *H. pylori* infection according to the manufacturer’s instructions. Participants who were eligible to have not taken antibiotics in the previous month and proton pump inhibitors at least two weeks before the test were included in this study.

### Definition of hyperuricemia

Due to the discrepancy between current guidelines and consensuses [26], hyperuricemia in this study was identified with the classic cut-off (> 6.0 mg/dL for females and > 7.0 mg/dL for males) [27] as well as with the cut-off of > 7.0 mg/dL for both males and females [28].

### Statistics analysis

All statistical analyses were performed using SPSS version 26.0 for Windows (SPSS Inc., Chicago, IL, United States). In the process of statistical analysis, continuous variables were presented as the mean and standard deviation (SD) or the median and interquartile range (IQR) as appropriate. The Student’s *t*-test or Mann-Whitney U-test was used for comparing continuous data. Categorical variables were presented as the percentage and compared by the χ2-test. After univariate analysis, factors with *P* < 0.05 were included in multivariate logistic regression. Multivariate logistic regression models were used to evaluate the risk factor for gout. In all statistical tests, a two-sided P-value < 0.05 was considered significant.

## Results

### Clinical and demographic characteristics of study subjects

This cross-sectional study involved 53,629 subjects, 31,692 males (59.1%) and 21,937 females (40.9%). The overall prevalence rate of *H. pylori* infection was 26.3% (27.6% in males and 24.4% in females). Hyperuricemia was identified in 15,797 subjects (720 subjects with gout and 15,077 subjects without gout).

Clinical characteristics of all participants as well as participants having hyperuricemia with/without gout are presented in Table 1. The gout group was older, had higher proportion of male gender, smoking percentage, drinking percentage, BMI, WC, SBP, DBP, FBG, HbA1c, ALT, AST, GGT, TG, Cr, UA levels and lower HDL-C levels than the hyperuricemia without gout group (*P* < 0.05). Notably, significantly higher *H. pylori* infection percentage was identified in the hyperuricemia subjects with gout than in those without gout (*P* < 0.001).

**Table 1:** Basic characteristics of the study participants with and without gout

Characteristics	Total (*n* = 53,629)	Hyperuricemia without gout (*n* = 15,077)	Gout (*n* = 720)	*t* value	*P*
Age (yr)	44.2 (12.0)	44.0 (12.4)	48.0 (11.5)	9.105	<0.001
Male gender (%)	59.1	81.2	98.5	139.127^a^	<0.001
Smoking (positive%)	22.9	30.2	42.5	48.886^a^	<0.001
Drinking (positive%)	32.8	44.3	56.8	43.686^a^	<0.001
*H. pylori* infection (positive%) (number)	26.3 (14,103)	27.2 (4108)	35.0 (252)	20.672^a^	<0.001
BMI (kg/m^2^)	23.9 (3.3)	25.5 (3.2)	26.5 (3.4)	8.156	<0.001
WC (cm)	82.8 (10.6)	88.3 (9.3)	92.4 (8.6)	12.130	<0.001
SBP (mmHg)	121.9 (16.7)	125.8 (16.0)	130.0 (15.7)	6.690	<0.001
DBP (mmHg)	73.2 (11.2)	76.1 (11.1)	79.6 (11.0)	8.216	<0.001
FBG (mmol/L)	5.17 (4.87–5.70)	5.27 (4.95–5.68)	5.42 (5.05–5.94)	6.769^b^	<0.001
HbA1c (%)	5.40 (5.20–5.70)	5.50 (5.20–5.80)	5.60 (5.33–6.60)	8.222^b^	<0.001
TP (g/L)	72.4 (4.0)	73.1 (4.0)	73.0 (4.1)	0.405	0.686
ALT (U/L)	20.0 (14.0–30.0)	26.0 (18.0-40.5)	29.0 (24.0–39.0)	3.559^b^	<0.001
AST (U/L)	21.0 (18.0–26.0)	24.0 (20.0–29.0)	25.0 (21.0–31.0)	4.389^b^	<0.001
ALP (U/L)	71.7 (21.7)	75.1 (20.7)	75.3 (19.7)	0.259	0.795
GGT (U/L)	22.0 (15.0–38.0)	33.0 (22.0–54.0)	42.0 (26.0–65.0)	7.498^b^	<0.001
TBil (µmol/L)	13.9 (11.0-17.8)	14.8 (11.8–18.6)	14.6 (11.6–18.6)	0.239^b^	0.811
TG (mmol/L)	1.25 (0.87–1.87)	1.69 (1.17–2.43)	2.07 (1.49–2.88)	9.744^b^	<0.001
TC (mmol/L)	5.08 (0.98)	5.25 (1.02)	5.28 (1.03)	0.783	0.434
LDL-C (mmol/L)	3.17 (0.79)	3.31 (0.81)	3.33 (0.82)	0.592	0.554
HDL-C (mmol/L)	1.31 (0.31)	1.20 (0.27)	1.16 (0.26)	3.873	<0.001
BUN (mmol/L)	5.02 (1.23)	5.26 (1.22)	5.33 (1.44)	1.416	0.157
CR (µmol/L)	72.2 (17.5)	80.3 (15.6)	89.7 (50.7)	4.951	<0.001
UA (µmol/L)	358.6 (92.0)	456.0 (429–540)	494.0 (417–561)	8.480^b^	<0.001

*Note* *Data are shown as mean (SD) or median (IQR) for continuous variables, shown as percentage of subjects (%) for categorical variables. a, χ2 value; b, Z value. *t* Value and *P* are presented as the results of comparisons between the hyperuricemia without gout group and the gout group. Hyperuricemia is defined as > 6.0 mg/dL for females and > 7.0 mg/dL for males. *Helicobacter pylori* (*H. pylori*), body mass index(BMI), waist circumference (WC), systolic blood pressure (SBP), diastolic blood pressure (DBP); fasting glucose (FBG), Glycated hemoglobin (HbA1c), low-density lipoprotein cholesterol (LDL-C), alanine aminotransferase (ALT), aspartate aminotransferase (AST), alkaline phosphatase (ALP), c-glutamyltransferase (GGT), total bilirubin (TBil), triglycerides (TG), total cholesterol (TC), low-density lipoprotein cholesterol (LDL-C), high-density lipoprotein cholesterol (HDL-C), urea nitrogen (BUN), creatinine (CR), and uric acid (UA)

### Risk factors analysis for gout in hyperuricemia subjects

Gender, age, smoking status, drinking status, *H. pylori* infection status, BMI, WC, SBP, DBP, FBG, HbA1c, ALT, AST, GGT, TG, Cr, HDL-C levels were entered into the original equation of the stepwise multiple regression model (Table 2). Model 1 entered serum uric acid (SUA) level as a covariate as well. Our results showed that age, male gender, drinking, WC, DBP, HbA1c, CR, UA, *H. pylori* infection were closely related to the risk for gout in hyperuricemia subjects in model 1. All the variables except for drinking status were also associated with the risk for gout in hyperuricemia subjects in model 2. Notably, *H. pylori* infection was found to be an independent risk factor for gout in both model 1 (OR: 1.320, 95%CI: 1.124–1.550; *P* = 0.001) and model 2 (OR: 1.327, 95%CI: 1.131–1.558; *P* = 0.001).

**Table 2:** Associated factors for gout in hyperuricemia population

Characteristics	Model 1 aOR (95%CI)	*P*	Model 2 aOR (95%CI)	*P*
Age	1.023 (1.016–1.030)	<0.001	1.021 (1.014–1.027)	<0.001
Male gender	8.366 (4.508–15.525)	<0.001	9.128 (4.917–16.916)	<0.001
Smoking	-	-	1.145 (0.975–1.344)	0.099
Drinking	1.194 (1.021–1.395)	0.026	1.167 (0.994–1.370)	0.059
WC	1.021 (1.012–1.031)	<0.001	1.024 (1.014–1.033)	<0.001
DBP	1.010 (1.003–1.017)	0.007	1.011 (1.004–1.018)	0.002
HbA1c	1.318 (1.208–1.437)	<0.001	1.306 (1.198–1.424)	<0.001
CR	1.011 (1.007–1.016)	<0.001	1.013 (1.009–1.018)	<0.001
SUA	1.003 (1.002–1.004)	<0.001	NA	NA
*H. pylori* infection	1.320 (1.124–1.550)	0.001	1.327 (1.131–1.558)	0.001

*Note* OR, odds ratio; CI, confidence interval; NA, not applicable. SUA was entered as a covariate in model 1. Hyperuricemia is defined as SUA > 6.0 mg/dL for females and > 7.0 mg/dL for males

### Association between H. *Pylori* infection and gout

Table 3 shows that *H. pylori* infection is significantly associated with the risk for gout in both univariate analysis (OR: 1.438, 95%CI: 1.229–1.683; *P* < 0.001) and multivariate analysis (OR: 1.320, 95%CI: 1.124–1.550; *P* = 0.001) after adjusting for confounding variables. When subjects were classified as hyperuricemia according to cut-off of > 7.0 mg/dL for both males and females, the results remains significant in both univariate analysis (OR: 1.422, 95%CI: 1.215–1.666; *P* < 0.001) and multivariate analysis (OR: 1.326, 95%CI: 1.130–1.557; *P* = 0.001).

**Table 3:** Odds ratio (OR) of *H. pylori* infection for gout in hyperuricemia population

	Unadjusted crude OR (95%CI)	*P*	aOR (95%CI)	*P*
Hyperuricemia 1	1.438 (1.229–1.683)	<0.001	1.320 (1.124–1.550)	0.001
Hyperuricemia 2	1.422 (1.215–1.666)	<0.001	1.326 (1.130–1.557)	0.001

*Note* aOR: Adjusted with age, gender, smoking, drinking, WC, DBP, HbA1c, CR and UA. Hyperuricemia 1 is defined as SUA > 6.0 mg/dL for females and > 7.0 mg/dL for males. Hyperuricemia 2 is defined as SUA > 7.0 mg/dL for both males and females

Table 4 shows the associated factors for gout in hyperuricemia population stratified by gender. *H. pylori* infection is closely associated with the risk for gout in male hyperuricemia subjects in multivariate analysis (OR: 1.326, 95%CI: 1.128–1.559; *P* = 0.001). In female group, however, *H. pylori* infection showed no significant association with the risk for gout (OR: 0.812, 95%CI: 0.205–3.216; *P* > 0.05).

**Table 4:** Associated factors for gout in hyperuricemia population according to gender

Characteristics	Male aOR (95%CI)	*P*	Female aOR (95%CI)	*P*
Age	1.023 (1.016–1.029)	<0.001	1.021 (1.014–1.027)	<0.001
Smoking	1.139 (0.969–1.338)	0.114	NA	NA
Drinking	1.160 (0.988–1.363)	0.070	1.079 (0.134–1.370)	0.059
WC	1.020 (1.010–1.030)	<0.001	1.056 (0.993–1.0122)	0.083
DBP	1.010 (1.003–1.018)	0.005	1.006 (0.949–1.066)	0.845
HbA1c	1.291 (1.181–1.411)	<0.001	2.253 (1.578–3.218)	<0.001
CR	1.012 (1.007–1.017)	<0.001	0.997 (0.950–1.047)	0.910
SUA	1.003 (1.002–1.004)	<0.001	1.000 (0.986–1.013)	0.943
*H. pylori* infection	1.326 (1.128–1.559)	0.001	0.812 (0.205–3.216)	0.767

Hyperuricemia is defined as SUA > 6.0 mg/dL for females and > 7.0 mg/dL for males

## Discussion

Our study provided evidence that *H. pylori* infection is significantly associated with the risk of gout in hyperuricemia population, particularly in men (OR, 1.430; 95% CI, 1.219–1.676). Multiple logistic regression analysis showed that gout individuals were older, with higher *H. pylori* infection percentage, drinking percentage, male gender percentage, higher levels of WC, DBP, HbA1c, Cr and UA comparing to asymptomatic hyperuricemia individuals. And *H. pylori* infection significantly contributed to the risk for gout under both of the two definitions of hyperuricemia.

Previous study found that half of individuals with serum urate level ≥ 10 mg/dL did not develop gout after 12-year follow up [29], implying other potential factors involved in the development of gout and crystal formation when tissue uric acid level is high. Our results indicated *H. pylori* infection as a risk factor for gout independent of SUA level under hyperuricemia condition. One of the possible explanations for the association between *H. pylori* infection and gout is that *H. pylori* infection may lead to a chronic inflammatory condition provoking the gout flare. Some studies found reduced circulating levels of C-reactive protein (CRP) after *H. pylori* eradication, despite the lack of verification in the sub-analysis of randomized controlled trials [30, 31]. Recent Mendelian randomization study indicated that *H. pylori* is associated with the risk of stroke, in which CRP may mediate the association [32]. Previous study demonstrated that the CRP level of gout patients is significantly higher than asymptomatic hyperuricemia patients, which is one of the most widely used inflammatory markers [33]. Another study indicated that oxidative stress and inflammatory markers including tumor necrosis factor, CRP, interleukin (IL) 1β, IL-6 may affect the development and clinical manifestations of gout based on hierarchical cluster analysis [34]. The priming and activation of the NLRP3 inflammasome may the potential mediating mechanism. The NLRP3 inflammasome relies on a two-signal initiation system, which avoids inappropriate activation of the pathway that might cause extra damage. In the gouty inflammation, the first signal results in stimulation of NF-κB via TLR4 and TLR2, and synthesis of pro-IL-1β and inflammasome components [35]. Monosodium urate crystals then act as the second activation signal, leading to the assembly of the inflammasome and activation of caspase-1, which cleaves pro-IL-1β to mature IL-1β^36^. IL-1β then triggers a signaling cascade resulting in the rapid recruitment of neutrophils and other cells to the site of crystal deposition and finally leads to the acute inflammation episodes [37]. While priming by the first signal is essential for inflammasome assembly in gout flare, this step is nonspecific and can be induced by multiple conditions or factors [6, 38]. Several studies have shown that *H. pylori* activated NLRP3 inflammasome in different cell types including dendritic cell, neutrophil, monocytic cell as well as macrophages [39–43]. The activation of TLR2 by bacterial factors has been indicated to mediate IL-1β production by regulating NLRP3 and pro IL-1β expression (signal 1) in H. *pylori* infected cells [41–44]. Therefore, chronic *H. pylori* infection is likely to contribute to the activation of NLRP3 inflammasome in gout flare by providing the first signal, which makes NLRP3 inflammasome a potential bridge between infections inflammation and non-infectious inflammation. Further studies may provide evidence for the mediating role of inflammatory cytokines between the relationship of *H. pylori* infection and gout.

The association between *H. pylori* infection and gout may also be mediated by gut microbiome. Gout patients showed less diverse gut microbiota than asymptomatic hyperuricemia patients, which may play a role in gout provocation [45]. Germ-free mice showed attenuated MSU crystal-induced inflammation and this effect was restored by reestablishing normal gut flora in murine model of gout [46]. There have been studies indicating that *H. pylori* may influence colonic microbiota both in animal experiments and clinical studies [47]. This effect of gut microbiota may participate in multiple *H. pylori*–associated extragastric diseases including gout.

Since SUA has long been credited as the key element to gout flare, most studies equated the influencing factors of SUA level with those of gout. Due to the high prevalence of hyperuricemia, screening the population with high-risk of gout can provide more precise indications for uric acid reduction therapy and reduce the medical burden. Therefore, our study focused on the hyperuricemia population and investigated factors associated to gout besides SUA level. SUA level of gout participants is significantly higher than asymptomatic hyperuricemia participants and independently associated with the incidence of gout flare. Our results also showed that older age, male gender, drinking habit, WC, DBP, HbA1c, CR and *H. pylori* infection were also associated with an increased risk of gout flare, independent of SUA. Previous study also indicated significant differences of age, serum creatine between gout and asymptomatic hyperuricemia groups [33], which is consistent with our study. The comorbid conditions of gout including metabolic syndrome, renal diseases and hypertension have been widely reported [48], accounting for the increase of waist circumference, diastolic blood pressure, HbA1c and creatine level indicated in our study. It has been widely acknowledged that the risk of gout was significantly higher in male gender as well as elderly people [7]. Though the male gender percentage of gout population (98.5%) in our study is higher than the recent epidemiological data in China [14], which is possibly due to the difference of age structure, economic conditions and lifestyles between health examination population and general population. People who take medical check-up tend to be relatively younger, making women more protected by the uricosuric action of oestrogen. While men with better economic conditions are more likely to have unhealthy diets. The result of gender subgroup analysis showed that older age and HbA1c were associated with higher risk of gout flare both for males and females. While *H. pylori* infection, WC, DBP, CR were significantly associated with higher risk of gout flare only for males, emphasizing the role of *H. pylori* infection for male patients with hyperuricemia. The mechanism behind the gender differences in the effect of *H. pylori* infection is yet unclear and needs to be further studied. Though the lack of adequate female patients with gout in this study may influence the reliability of the result of female subgroup analysis, warranting for further investigation.

Results from the current study should be interpreted in consideration of several limitations. First, whether *H. pylori* infection is a bystander, a causal factor or a consequence of gout cannot be answered due to the cross-sectional study design. Since most of the *H. pylori* infections are acquired in childhood long before the onset of gout and persist throughout life [49], *H. pylori* infection is more likely to play a role in the development of gout. Besides, we speculate that the gout rate might be underestimated due to the self-reported method. Third, other underlying confounding variables including *H. pylori* eradication history, medication history like UA lowering therapy, diuretics that might influence the SUA, environmental factors like economic status, low purine diet were lacking due to the retrospective study design. The menopause age for female subjects was in lack, which is an important factor that impact SUA. Yet we conducted the analysis based on two definition of hyperuricemia and the results remained to be significant. And the physical examiners who voluntarily self-reported a history of *H. pylori* eradication were excluded in the study to minimize the bias. Last but not least, UBT was used to confirm *H. pylori* infection status without further definitive examination including histology or *H. pylori* culture in our study. Nevertheless, UBT is recommended as the best non-invasive approach to the diagnosis of *H. pylori* infection [50], since most previous studies indicated a similar high sensitivity and specificity ranging from 90 to 100%^51^. Prospective studies with a large population are warranted to further explore the correlation between *H. pylori* infection and gout especially in hyperuricemia population.

## Conclusion

In conclusion, our study showed a significant correlation between *H. pylori* infection and gout, especially in men. Further studies exploring the role of *H. pylori* in gout flare will not only expand our understanding of the mechanism of gout, but also contribute to the development of new prevention and treatment strategies for the disease.

## Data Availability

The raw data supporting the conclusions of this article will be made available by the authors, without undue reservation.
